# Diabetes, periodontitis, and cardiovascular disease: towards equity in diabetes care

**DOI:** 10.3389/fpubh.2023.1270557

**Published:** 2023-12-21

**Authors:** Constanza Serón, Pablo Olivero, Nicolás Flores, Benjamín Cruzat, Francisca Ahumada, François Gueyffier, Ivanny Marchant

**Affiliations:** ^1^Laboratorio de Modelamiento en Medicina, Escuela de Medicina, Universidad de Valparaíso, Valparaíso, Chile; ^2^Clinical Studies Unit, Escuela de Medicina, Universidad de Valparaíso, Valparaíso, Chile; ^3^Laboratorio de Estructura y Función Celular, Escuela de Medicina, Facultad de Medicina, Universidad de Valparaíso, Valparaíso, Chile; ^4^Laboratoire de biologie et biométrie évolutive – équipe modélisation des effets thérapeutiques, Université Claude Bernard Lyon, Lyon, France

**Keywords:** type 2 diabetes mellitus, periodontitis, physicians and dentists, systemic diseases, inequality, inflammation, periodontal disease

## Abstract

Type 2 diabetes and its associated cardiovascular risk is an escalating epidemic that represents a significant public health burden due to increased morbidity and mortality, disproportionately affecting disadvantaged communities. Poor glycaemic control exacerbates this burden by increasing retinal, renal, and cardiac damage and raising healthcare costs. This predicament underscores the urgent need for research into cost-effective approaches to preventing diabetes complications. An important but often overlooked strategy to improve metabolic control in diabetic patients is the treatment of periodontitis. Our aim is to assess whether the inclusion of periodontitis treatment in diabetes management strategies can effectively improve metabolic control, and to advocate for its inclusion from an equity perspective. We conducted a comprehensive review of the literature from 2000 to 2023. We analyzed the pathophysiological links between periodontitis, diabetes, and atherosclerotic cardiovascular disease, all of which have inflammation as a central component. We also examined the inequalities in health care spending in this context. Our findings suggest that incorporating routine screening and treatment of periodontitis into national health programs, with coordinated efforts between physicians and dentists, is a cost-effective measure to improve metabolic control, reduce complications and improve the overall quality of life of people with diabetes.

## Introduction

Type 2 diabetes (T2DM) is one of the fastest-growing global diseases of the 21st century, with a prevalence that has more than tripled in the last 20 years and affects 10.5% of the world’s population aged 20–79 years (1). It is expected that these numbers will continue to grow, particularly in low-income countries, which have higher population growth compared to high-income countries. T2DM also generates a large impact on indirect health expenditure, i.e., workforce dropout, absenteeism and presenteeism (2), affecting the poorest countries, which account for the lowest percentage of global diabetes-related health expenditure but have the largest outflow from the national health budget attributed to T2DM (1, 2). This picture becomes even more striking when we consider that half of the people diagnosed with diabetes in the United States achieve a glycosylated hemoglobin (HbA1c) < 7% (3), one of the markers with the highest predictive value for microvascular and macrovascular diabetes complications (4), with a 1% reduction in HbA1c being able to reduce the risk of any diabetes-related endpoint by 21% (5). More worryingly, only 1 in 4 or fewer adults with T2DM meet the American Diabetes Association (ADA) targets for blood pressure, cholesterol, and HbA1c to reduce their cardiovascular risk (6, 7). Failure to meet these targets results in a significant burden of disability and death (8), which has contributed to a tripling of global health expenditure on diabetes over the past 15 years (1), disproportionately affecting the poorest regions (1, 2). Much of this expenditure is attributable to atherosclerotic cardiovascular disease (ACVD), defined as coronary heart disease, cerebrovascular disease, or peripheral arterial disease of presumed atherosclerotic origin. ACVD is the leading cause of morbidity and mortality in people with diabetes, and the current paradigm of diabetes care requires that multiple cardiovascular risk factors be addressed simultaneously and aggressively (6).

This scenario implies a constant need to investigate new cost-effective methods to provide antidiabetic therapy and reduce T2DM cardiovascular risk and complications. An important but often neglected measure in the T2DM management is the treatment of periodontal disease (PD) (9). PD is a group of chronic inflammatory diseases that affect the tooth supporting structures and is classified into gingivitis, the mildest form of the disease, characterized by swollen, red and easily bleeding gums without loss of underlying alveolar bone, and periodontitis, an advanced state of the disease that involves both inflammatory changes and destruction of the periodontal ligament and supporting alveolar bone, which can lead to tooth loss if left untreated (10). Severe periodontitis has a worldwide prevalence of 5%–15% in the adult population (10) and, compared to other dental diseases, it has the highest correlation with chronic systemic diseases, especially T2DM. In diabetic patients, periodontitis is known to compromise glycemic control and perpetuate chronic inflammation, increasing the risk of microvascular and macrovascular complications (11).

In this article, we will review the pathophysiological and social links between PD and T2DM, focusing on the evidence for the benefits of periodontal treatment in controlling glycaemia, inflammatory markers, and cardiovascular risk. Our aim is to assess whether incorporating periodontal treatment into diabetes management can effectively improve metabolic control, with a significant impact on cardiovascular outcomes and the overall quality of life of patients with diabetes, with a particular focus on disadvantaged communities. We conducted a comprehensive review of the PubMed literature between 2000 and 2023 using relevant terms, PubMed functions, and citation tracking. Given the substantial growth in research on periodontitis and diabetes over the past 15 years, priority was given to recent literature, with particular attention to key studies on the systemic impact of periodontitis, standards of care, oral health management, and systematic reviews.

### Inequality and neglect in oral health care: uncovering disparities and urging action

It is estimated that 90% of the world’s population will suffer from some form of oral disease during their lifetime. In 2010, five oral diseases alone accounted for 18,814,000 disability-adjusted life years (DALYs), an increase of 21% since 1990 (10). This increase in DALYs is significantly higher for PD, which has risen by 57% in 20 years to become the leading oral disease in adults over the age of 35, along with other major non-communicable diseases (NCDs), such as diabetes (69.0%) (10). In addition, dental caries and PD are the leading causes of tooth loss and edentulism worldwide, resulting in difficulties with chewing, food intake, speech, self-esteem, quality of life and social interactions (10, 12). These conditions also hinder economic development. In Canada, more than 40 million hours are lost each year due to dental problems and treatment, representing a potential productivity loss of more than $1 billion (13).

Due to the high prevalence and the cumulative and recurrent nature of PD and other oral diseases, oral health care is one of the most expensive services provided by health systems; thus, with average annual expenditure in the European Union between 2008 and 2012 of 79 billion euros (€), exceeding the costs of stroke (€38.0 billion), cancer (€51.0 billion) and respiratory diseases (€55.0 billion) (10). However, the way in which governments address oral health varies widely between countries and among their own populations, with notable inequalities. A 2016 multinational comparison estimated the extent of social inequality in adult oral health in five countries, using education as an indicator of social position (12). Of the five Organization for Economic Cooperation and Development countries, the richest country, the United States, had the highest prevalence of edentulism at almost all levels of education, with high inequality, while in the poorest country, Chile, more than half of the population with the lowest level of education lacked functional dentition compared with 10% of the university population (12, 14).

Worldwide, several countries and health systems report a lack of oral health coverage and inequitable access, implying a significant impoverishment of communities due to out-of-pocket health expenditure (15, 16). Bernabé et al. (15) analyzed the impact of out-of-pocket dental health expenditure on catastrophic health expenditure (CHE) in households in low- and middle-income countries. They found that households that paid for dental care were more likely to experience CHE or impoverishment than those that did not pay for dental care. At the country level, of all the covariates analyzed, higher out-of-pocket health expenditure was the only factor associated with greater odds of both CHE and impoverishment (2).

Developed countries have developed strategies to address this problem (16–18), but in Latin America, only five governments have even assessed dental status in nationally representative samples of adults (19). The neglect of oral health, particularly in low- and middle-income countries, represents a major blind spot for public policies aimed at addressing NCD epidemics, as the relationship between dental conditions and other NCDs has been extensively studied, particularly PD, the dental condition with the strongest observed association with systemic diseases, particularly T2DM (20).

### Impact of periodontitis on diabetes and cardiovascular risk

**Figure 1:**
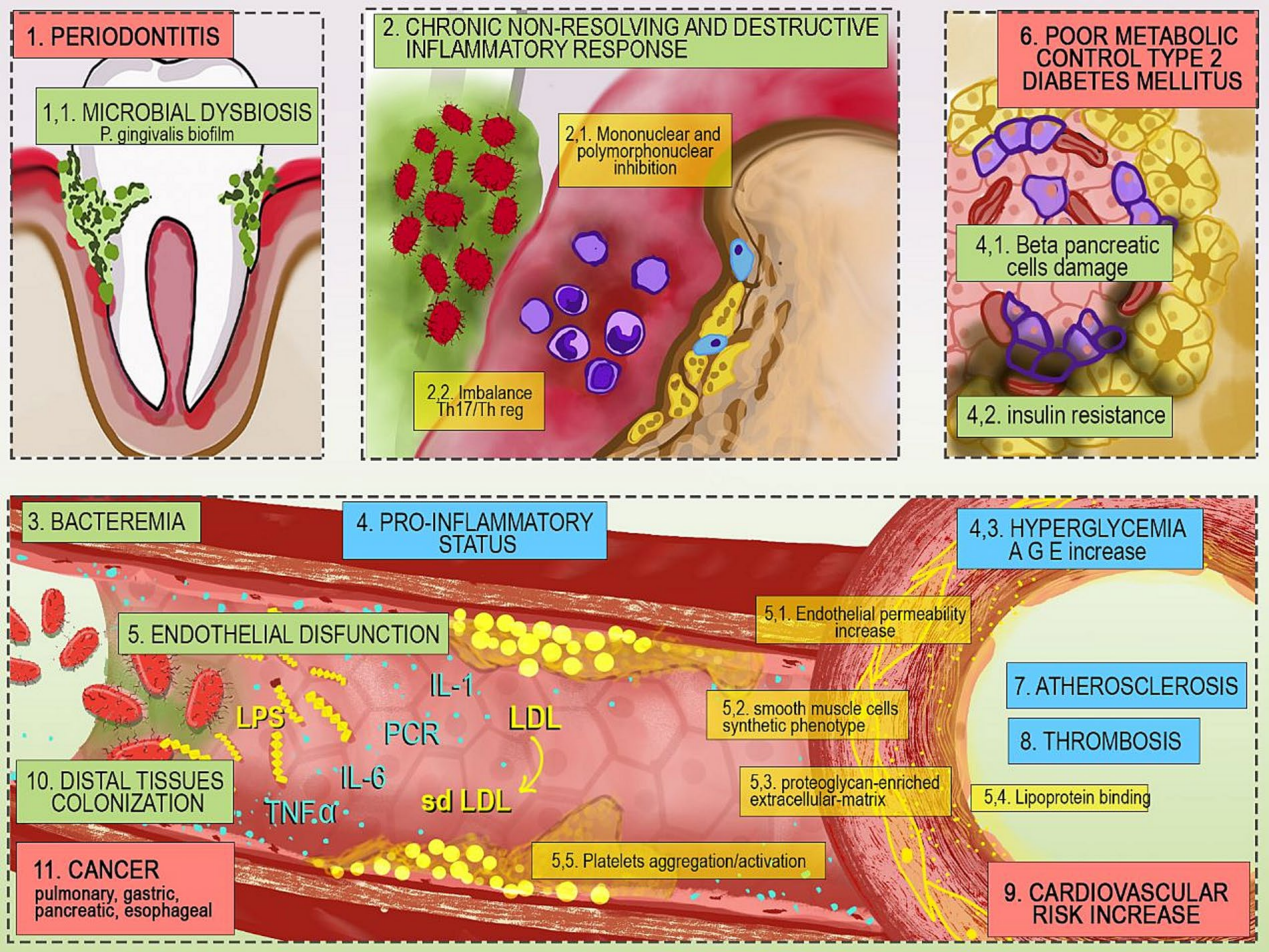
Mechanisms underlying the relationship between periodontitis and systemic diseases. 1. Microbial dysbiosis favors proliferation of *P. gingivalis*; 2. Bacterial infection destroys connective tissue and alveolar bone allowing the entry of various microbes into the bloodstream 3. The gram-negative pathogen *P. gingivalis* activates a pro-inflammatory status that 4. impairs pancreatic function and glycemic control; 5. The resulting endothelial dysfunction, combined with the action of bacterial endotoxins worsens 6. metabolic control and contributes to 7. Atherosclerosis, 8. Thrombotic phenomena and 9. Increased cardiovascular risk. 10. In addition, endothelial dysfunction allows colonization of distal tissues and 11. Increased risk of cancer of the lung, pancreas, esophagus, and stomach. Th17, T helper 17 lymphocyte; Threg, regulatory T helper lymphocyte; LPS, lipopolysaccharide; IL-6, interleukin 6; TNF-α, tumor necrosis factor alpha; PCR, C-reactive protein; IL-1, interleukin 1; sdLDL, small and dense low-density lipoprotein; LDL, low-density lipoprotein; AGE, advanced glycation end products.

In 2012, the Joint EFP/AAP Workshop on Periodontitis and Systemic Disease concluded that there is consistent and strong epidemiological evidence that periodontitis increases the risk of ACVD (21), but the quality of the evidence at that time did not allow causality to be inferred (22). In addition, diabetic patients with periodontitis have been shown to have a 6-fold higher risk of poor glycemic control and an increased risk of diabetic complications than patients with a healthy periodontium (11, 23). The mechanisms underlying these associations have been attributed to the relationship between local and systemic inflammation (24, 25) (Figure 1).

PD is initiated by the accumulation of pathogenic dental biofilm around the gingival margin (11). Diabetic patients have a 2–3 times higher risk of PD than non-diabetics, especially those with inadequate glycemic control (26), as chronic hyperglycemia and the accumulation of advanced glycation end products (AGEs) increase cytokine expression and oxidative stress in mononuclear and polymorphonuclear phagocytic cells (27), create a pro-inflammatory environment with high levels of serum concentrations of C-reactive protein (CRP), IL-1, IL-6, and TNF-α, and reduce both collagen synthesis and osteoblastic activity. These conditions facilitate the transformation of the subgingival microflora into an essentially high-risk gram-negative flora, especially with *Porphyromonas gingivalis* (*P. gingivalis*), generating dysbiosis and a chronic non-resolving and destructive inflammatory response on the connective tissue and alveolar bone, facilitating the entry of microbes into the bloodstream, and cause bacteremia (27, 28). *P. gingivalis* endotoxins (lipopolysaccharides – LPS) induce systemic responses. LPS facilitate the three critical steps in the pathogenesis of atherosclerosis: (1) increased levels of small and dense low-density lipoprotein (sd-LDL); (2) promotion of endothelial permeability by triggering the innate immune system response; and (3) stimulation of smooth muscle cells to produce a proteoglycan-enriched extracellular matrix in the intima that enhances lipoprotein binding and retention (29). It has also been shown that certain strains of *P. gingivalis* induce platelet activation and aggregation even in the absence of vascular injury, favoring thrombotic episodes (30, 31). In addition, the chronic pro-inflammatory response stimulated by LPS through the ulcerated bursal epithelium may reduce beta-cell function, increase insulin resistance and worsen dyslipidemia, creating a cycle of hyperglycemia and AGE protein binding, which reinforces the diabetic pathway of connective tissue degradation, destruction, and proliferation, leading to more frequent and severe PD in people with diabetes, increased difficulty in controlling blood glucose and increased risk of ACVD (11, 27, 32).

### Improving periodontitis management and metabolic control in individuals with diabetes

In addition to pharmacological treatment, the World Health Organization recommends a healthy diet, physical activity, and regular screening and treatment for complications to prevent adverse outcomes (33). In this context, any measure to improve glycemic control is welcomed.

The ADA concludes in its 2022 edition of the Standards of Medical Care in Diabetes, that the benefits of periodontal treatment in diabetic patients remain controversial (9) because, although the evidence clinically and statistically demonstrates a reduction in HbA1c in T2DM ranging from 0.27% to 0.48% at 3–4 months post-intervention with a reduction similar to that usually achieved by adding a second anti-diabetic agent to the pharmacological regimen (11, 34), and also comparable to the expected advantage from the insulin pen over the classical syringe method of administration in insulin users (35); the Cochrane review on this topic in 2015, rated this body of evidence as weak and insufficient to demonstrate that this effect could be sustained beyond 4 months (34). Since then, new evidence has been published. In 2018 a randomized clinical trial (RCT) by D’Aiuto et al. (36) showed that intensive and standardized periodontal treatment of moderate-to-severe periodontitis in adults with T2DM achieved a sustained 0.6% reduction in HbA1c after 12 months of follow-up. In 2021, new evidence (37) emerged on the efficacy of adding an adjunctive drug to non-surgical periodontal treatment with extended follow-up beyond 4 months. This RCT achieved a > 1% reduction in HbA1c at 3 and 6 months, with or without adjunctive antibiotic prophylaxis (37). The most recent Cochrane review in 2023 thus modified its conclusions, indicating that periodontal treatment with subgingival instruments improves glycemic control over 6 months in patients with both diabetes and periodontitis by a clinically meaningful proportion compared with no treatment or usual care, with moderate certainty of evidence (38), consistent with recent systematic reviews (39).

### Periodontal treatment: a catalyst for inflammation control

In 2017, the CANTOS trial (40) showed that reducing vascular inflammation significantly reduced the rate of recurrent cardiovascular events even in the absence of concomitant lipid lowering. The main indicator of the reduction in inflammation was CRP, a known prognostic marker of vascular events associated with various metabolic conditions (29). In addition to the improvement in glycemic control, D’Aiuto et al. also confirm a reduction in inflammatory markers such as CRP and TNF-α after 6 and 12 months of follow-up with a lower 10-year risk of coronary heart disease, consistent with evidence of a direct association of periodontal therapy with lower rates of myocardial infarction and heart failure in T2DM (26). This improvement in the overall inflammatory status is also the proposed mechanism to explain the reduction in creatinine levels in patients with type 2 diabetes receiving intensive periodontal therapy (36, 41) and the lower incidence rate of end-stage renal disease in the general population following surgical treatment of periodontitis (42).

### Critical considerations for public health policy: integrating periodontal treatment into diabetes management

The bidirectional relationship between PD and T2DM has long been recognized, to the extent that the recent EFP/AAP World Workshop proposed the inclusion of glycemic control in the diagnostic scheme for patients with periodontitis (43). While hyperglycemia increases the risk and severity of PD, periodontitis worsens metabolic control in T2DM (11, 26). Consistent with this evidence, a USA study published in 2009 found that using periodontal measures to assess T2DM risk significantly improved screening rates for prediabetes and diabetes (44), and early detection led to recommendations for cost-effective lifestyle changes that resulted in a significant proportion of patients with prediabetes normalizing their blood glucose levels (45). These results encouraged NICE in the UK to recommend the involvement of dentists and physicians in screening for T2DM (11).

On the other hand, PD is a highly relapsing chronic disease, in which inflammation promotes the growth of dysbiotic microbial communities of “inflammophilic” bacteria that perpetuate tissue destruction in a vicious cycle of mutually reinforcing dysbiosis and inflammation (46, 47). Therefore, a key element of therapeutic success is to restore a healthy and durable oral ecosystem with long-term stability to favor improvement in clinical parameters such as probing depth, bleeding on probing or suppuration (46), and inflammatory parameters, to control both dysbiosis and disease progression (47). Thus, regular examination and treatment of recurrent PD would likely contribute to metabolic control and stability in patients with T2DM. Experts have stated that asking about symptoms and performing a visual assessment of oral and periodontal changes is straightforward and should be part of the evaluation of diabetic patients to make the necessary referrals (9, 45).

The oral health community has led several initiatives as screening tools. In a pilot study in the US, chairside HbA1c testing was administered to 50 patients with periodontitis who reported one or more of the ADA’s T2DM risk factors, detecting 32% of individuals with pre-diabetic HbA1c levels. Each HbA1c test costs US$9 (48). A more affordable option was demonstrated in Germany, where the Find-Risk questionnaire was modified to add the presence or absence of severe periodontitis and administered to general patients attending private dental services, with a positive predictive value of 35%. They were referred to a diabetologist for further diagnosis, where more than half of the patients had altered blood tests (49). Table 1 summarizes some recommendations for clinicians assessing patients with diabetes and dentists assessing adults with PD.

**Table 1:** Recommendations for physicians and dentists for screening and prevention of T2DM and PD.

**Medical assessment of diabetes to screen PD**		
**Immediate referral to dentist**	**Recommend professional oral examination**	**Prevention of PD**
Prior diagnosis of PD with no or poor follow-up of care.	Annual periodontal check-up for all persons with newly diagnosed diabetes mellitus, especially smokers.	Education: About the increased risk of PD, and the importance of watching for any signs or symptoms of gum disease.
Signs or symptoms of PD: Bleeding gums during brushing or eating. Loose teeth Spacing or separation of the teeth Bad oral odor and/or bad taste Abscesses in the gums or gingival discharge.	Common oral problems in patients with diabetes: Dry mouth Burning mouth Poor healing of oral wounds Candida infections	Action: Advise that successful periodontal therapy can improve glycemic and metabolic control. Promote proper oral hygiene, i.e., twice-daily brushing for a minimum of 2 min, inter-dental cleaning, etc.
**Dental assessment of patients with PD to screen T2DM**		
**Immediate referral to physician**	**Recommend testing for prediabetes and T2DM**	**Prevention of T2DM**
Prior diagnosis of T2DM with little or no follow-up care. Ask about blood glucose and HbA1c levels.	Overweight or obese adults with one or more risk factors:	Education: About the increased risk of T2DM, especially in the presence of other risk factors.
In the presence of classical symptoms, such as: Polydipsia Polyuria Polyphagia Unexplained weight loss	First-degree relative with diabetes History of ACVD, use of anticoagulants or antiplatelet agents Hypertension Dyslipidemia or users of lipid-lowering drugs Women with polycystic ovary syndrome Physical inactivity Conditions associated with insulin resistance, i.e., acanthosis nigricans, skin tags, etc.	Action: Referral to primary care, diabetes prevention program and/or nutrition programs available in your country.

### Inequalities in periodontal treatment and diabetes

There are several barriers to integrating PD prevention, control, and treatment into diabetes care programs. First, the current model of Westernized modern dentistry with a strong focus on treatment and high technology (50) greatly increases oral health expenditures, making it difficult for low- and middle-income countries to cover oral health services and increasing out-of-pocket expenditures for vulnerable populations, who are at greater risk of CHE and impoverishment (15). The prevalence of T2DM is directly related to lower socioeconomic status, less access to healthy food, lack of safety, and lower educational attainment in different countries (51). On the other hand, a low educational attainment has been shown to be a predictor of chronic PD, a relationship that is partly explained by the association between the educational attainment and T2DM (52), in addition to their common risk factors such as smoking, age, gender and overweight (20). A second barrier is the significant recruitment bias that exists in clinical studies of the effect of periodontal treatment on HbA1c, where the predominant participants are middle-aged white women with access to primary care providers, making it difficult to extrapolate results to those most at risk for PD and T2DM: older men, low socio-economic status, and racial/ethnic minorities (53).

In Latin America, Chile is one of the five countries that have assessed dental status in nationally representative samples of adults (19). In the early 2000s, this country embarked on a major health reform that included oral health, with the aim of improving access to and quality of health care and reducing health inequalities (54). Currently, inequalities in dental service utilization and untreated dental caries have been reduced. However, remaining teeth inequalities have increased, which can be explained by the application of low-complexity treatments with frequent dental extraction in low-income adults with free access to dental care (54). In addition, the Chilean health reform was designed to target the most prevalent diseases and at-risk populations. As a result, dental programs benefit certain age groups and pregnant women but ignore other populations with a high prevalence of oral disease, such as those with cardiovascular disease, diabetes or other NCDs (55).

## Discussion

Both T2DM and PD are major causes of illness that can lead to disability and death (1, 2, 10). These conditions disproportionately affect low- and middle-income countries (12, 16). PD is two to three times more common in people with diabetes than in the general population (26). It is associated with a 6-fold higher risk of poor glycemic control and increased risk of complications compared with diabetic patients with a healthy periodontium (29). The effect of periodontal treatment on reducing HbA1c is comparable to the effect of adding a second drug to the antidiabetic pharmacological regimen (11). Despite historical controversy regarding the benefits of treating periodontitis in diabetes (9), recent evidence clearly confirms its benefits in terms of glycemic control, renal function, cardiovascular risk reduction, alleviation of chronic inflammation, and improvement in quality of life (36, 56); all with a clinically significant impact (38).

In addition, prevention of PD in T2DM may be a cost-effective strategy, involving regular check-ups in which clinicians inquire about symptoms and perform oral visual assessments (9, 11, 46). On the other hand, dentists can play an important role in the screening of T2DM through earlier medical evaluation in prediabetic stages (48, 49). Based on the evidence reviewed, it is also important to discuss the need to expand the services covered by T2DM insurance plans. This could make screening and improvement of glycemic status through PD treatment affordable in low- and middle-income countries.

Strengths of our study include a comprehensive review of the literature across multiple domains and the potentially high impact of our findings, given the widespread prevalence of both T2DM and PD. However, it is important to acknowledge its limitations. The certainty of our findings is limited by the exploratory nature of this review, which differs from the rigor of a systematic review. In future phases, our agenda includes evaluating the efficacy and safety of readily implementable interventions by both physicians and dentists to improve standards of care in the outpatient setting.

We must keep in mind the relapsing nature of PD and that durable metabolic control in T2DM may depend not only on the success of PD treatment, but also on regular follow-up to re-establish a healthy and durable oral ecosystem with long-term stability to favor improvement in clinical parameters and keep the inflammatory state in check. Finally, the prevention, treatment and control of PD in diabetic patients should be included in public health policies to improve metabolic control and quality of life and reduce diabetes complications not only in high-income patients but also in the most socially disadvantaged patients.

## Author contributions

CS: Investigation, Validation, Writing – original draft. PO: Investigation, Validation, Writing – original draft. NF: Investigation, Validation, Visualization, Writing – original draft. BC: Investigation, Validation, Visualization, Writing – original draft. FA: Investigation, Validation, Visualization, Writing – original draft. FG: Conceptualization, Supervision, Validation, Writing – review & editing. IM: Conceptualization, Investigation, Methodology, Supervision, Validation, Writing – original draft, Writing – review & editing.
